# Transforaminal epidural injection versus continued conservative care in acute sciatica (TEIAS trial): study protocol for a randomized controlled trial

**DOI:** 10.1186/s12883-019-1445-9

**Published:** 2019-09-03

**Authors:** Eduard Verheijen, Alexander G. Munts, Oscar van Haagen, Dirk de Vries, Olaf Dekkers, Wilbert van den Hout, Carmen Vleggeert-Lankamp

**Affiliations:** 1 Department of Neurosurgery, Leiden University Medical Center, Albinusdreef 2, PO Box 9600. 2300, RC Leiden, The Netherlands; 2 Department of Neurology, Spaarne Gasthuis, Haarlem, /Hoofddorp, The Netherlands; 3 Department of Anaesthesiology, Spaarne Gasthuis, Haarlem, /Hoofddorp, The Netherlands; 4 Department of Anaesthesiology, Spaarne Gasthuis, Haarlem, /Hoofddorp, The Netherlands; 5 Department of Epidemiology, Leiden University Medical Center, Leiden, The Netherlands; 6 Department of Biomedical Data Science, Leiden University Medical Center, Leiden, The Netherlands; 7 Department of Neurosurgery, Leiden University Medical Center, Leiden, The Netherlands

**Keywords:** Acute sciatica, Herniated intervertebral disk, Lumbar spine, Transforaminal epidural injection, Randomized controlled trial, Cost-effectiveness, Prolonged conservative treatment, Leg pain, Lumbar surgery

## Abstract

**Background:**

Sciatica is a condition that is characterised by radicular pain in the leg and primarily caused by a herniated lumbar intervertebral disk. In addition to leg pain, patients can experience back pain, leg numbness and leg weakness resulting in decreased productivity and social activity. The majority of sciatica cases recovers spontaneously and therefore patients are initially treated conservatively with oral pain medication. However, some patients experience intractable pain that severely impedes them and no consensus exists on the optimal conservative treatment to reduce this discomfort in the acute phase of sciatica. The aim of the TEIAS trial is to assess the effectiveness, cost-effectiveness and predictive capability on patient outcome of transforaminal epidural injection (TEI) compared to treatment with standard pain medication.

**Methods:**

This study is designed as a prospective, open-label, mono-centered, randomized controlled trial. Patients that visit their general practitioner with complaints of radicular leg pain and meet the selection criteria are asked to participate in this study. Eligible patients will be randomized to treatment with TEI or to treatment with standard oral pain medication. Treatment of TEI will comprise lidocaine with methylprednisolone acetate for L3 and below and lidocaine with dexamethasone above L3. A total of 142 patients will be recruited and follow-up will occur after 1, 2, 4, 10 and 21 weeks for assessment of pain, functionality, patient received recovery and cost-effectiveness. The primary outcome will be the average score for leg pain at 2 weeks. For this outcome we defined a clinically relevant difference as 1.5 on the 11-point NRS scale.

**Discussion:**

Adequate conservative treatment in the acute phase of sciatica is lacking, particularly for patients with severe symptoms. Focusing on effectiveness, cost-effectiveness and predictive capability on patient outcome of TEI will produce useful information allowing for more lucid decision making in the conservative treatment of sciatica in the acute phase.

**Trial registration:**

This trial is registered in the ClinicalTrials.gov database under registry number NCT03924791 on April 23, 2019.

## Background

Sciatica is a condition that is characterised by radicular pain in the leg and generally caused by a herniated lumbar intervertebral disk. The herniated disk exerts pressure onto a lumbar nerve root that follows its way into the sciatic nerve. In addition to leg pain, this can lead to back pain, muscle weakness or muscle numbness resulting in decreased productivity and social activity [1–5]. This condition is regularly reported in GP practices and a lifetime prevalence around 40% has been recorded [6]. Together with other back pain-related conditions sciatica has a vast impact on the economy. In 2007, total costs of patients with lower back pain were estimated to be 3.5 billion euros in the Netherlands of which 88% was contributable to absenteeism and loss of productivity [7].

The prognosis of sciatica is favourable since the majority of cases recovers spontaneously. Therefore, patients are initially treated conservatively with standard oral pain medication unless cauda equine syndrome or progressive paresis is reported. Those groups of patients are directly referred to the neurologist or neurosurgeon. An earlier RCT demonstrated that surgical therapy resulted in a similar outcome compared to conservative therapy at 26 weeks follow-up and 40% of the patients assigned to the conservative group crossed over to the surgical group with a mean delay of 15 weeks [8]. Due to this knowledge it is now common to only offer surgery after 8 weeks of conservative care and preferably extend this time period to a total of 14–16 weeks in a process of Shared Decision Making [9, 10]. After this period of ‘wait-and-see’ patients can opt for surgery if symptoms have not ameliorated sufficiently. However, during the first weeks it can be challenging to sufficiently control the patient’s pain and adequate treatment to reduce this discomfort in the acute phase of sciatica is yet to be found.

Transforaminal epidural injections (TEI) therapy might be the answer as it is postulated to have an analgesic and anti-inflammatory effect which can last several days to weeks. Analgesics and/or corticosteroids are deposited through a transforaminal approach in close proximity of the affected nerve root. Theoretically, this treatment would enable the patient to remain physically active while awaiting spontaneous recovery. Although its use is widespread, some patients experience a beneficial effect while others report no effect at all or only initial pain relief followed by recurrence of pain after several hours, days or weeks [11, 12]. In the Netherlands some hospitals offer TEI as standard care for sciatica but usually only after 14–16 weeks of conservative therapy.

Due to limited literature on TEI in patients with acute sciatica, it is still unclear whether TEI is a more (cost-)efficacious type of conservative treatment than oral pain medication in the acute phase. It remains difficult to differentiate between responders and non-responders and suggestions that response to TEI can be a predictor for long-term patient outcome require further research [12–14]. This study has the potential to answer relevant questions about TEI as minimally invasive conservative treatment in acute sciatica, assessing effectiveness on the short term and long term, predictive capability and cost-effectiveness.

## Design and methods

### Study design

The TEIAS (Transforaminal Epidural Injection in Acute Sciatica) Trial is designed as a prospective, open-label, mono-centred, randomized controlled trial. Eligible patients will be randomized to treatment with TEI (intervention group) or to treatment with standard oral pain medication (control group). Patients will be followed for a total of 21 weeks.

### Aims and objectives

The aim of the TEIAS Trial is to determine whether transforaminal epidural injections are a more efficacious treatment to alleviate symptoms in patients suffering from acute sciatica than the current standard treatment with oral pain medication.

The primary objective is to estimate:

1. The average score on leg pain at 2 weeks after treatment with TEI compared to usual conservative care.

Secondary objectives are to estimate:

2. The duration of effect from TEI;
3. The percentage of patients that have experienced a satisfactory decrease in leg pain and increase in functionality at 4 weeks after treatment with TEI compared to usual conservative care;
4. The response to TEI at 2 weeks as a predictor of leg pain at 14–16 weeks and 26 weeks after onset of symptoms;
5. The correlation between response in leg pain, functionality and patient satisfaction at 1 week and at 2 weeks after randomization and at 10 and 21 weeks after randomization;
6. The correlation between baseline data and responders and non-responders to TEI after 2 weeks;
7. Cost-effectiveness of TEI to alleviate symptoms for acute sciatica;

### Study setting and patient recruitment

This study will take place at the Spaarne Gasthuis (SG), Hoofddorp and Haarlem, the Netherlands in cooperation with researchers from the Department of Neurosurgery, Leiden University Medical Center (LUMC), Leiden, the Netherlands. Patient recruitment will occur at general practitioner offices that are part of the GP collective Haarlemmermeer and Kennemerland which embodies a total of 185 GP’s. Patient inclusion will continue until the target sample size has been reached.

### Patient enrolment

Patients suffering from acute sciatica that visit their general practitioner will be referred for participation in this study if they have a Numerical Rating Score (NRS) for leg pain of 6 or more on a 11-point scale and symptom duration of 3–8 weeks. Patients are contacted by the study investigator and inclusion and exclusion criteria are checked to determine whether the patient is eligible. Furthermore, it will be ascertained that the patient has access to e-mail in order to fill in the digital questionnaires at home. If patients are willing to cooperate and submit their informed consent to the researcher, they are referred to the neurosurgeon. The neurosurgeon will examine the patient and if the neurosurgeon is convinced that the patient is suffering from sciatica, the patient is enrolled by the researcher and randomized to receive TEI from the anaesthesiologist or to continue usual conservative care (oral pain medication). A complete description of inclusion and exclusion criteria can be found in Table 1.

**Table 1:** Patient inclusion and exclusion criteria

Inclusion criteria	Exclusion criteria
Diagnosed with sciatica by GP	Age under 18 years or above 55 years
NRS leg pain of 6 or more points on a 11-point scale with a duration of > 3 and < 8 weeks	Condition preventing to receive TEI
Confirmation of sciatica by neurosurgeon	Severe scoliosis
Signed informed consent	TEI received in 6 months before randomization date
	Surgery for sciatica at the same level
	Surgery for sciatica at another level within one year before inclusion
	Pregnancy

### Description of processes

**Fig. 1:**
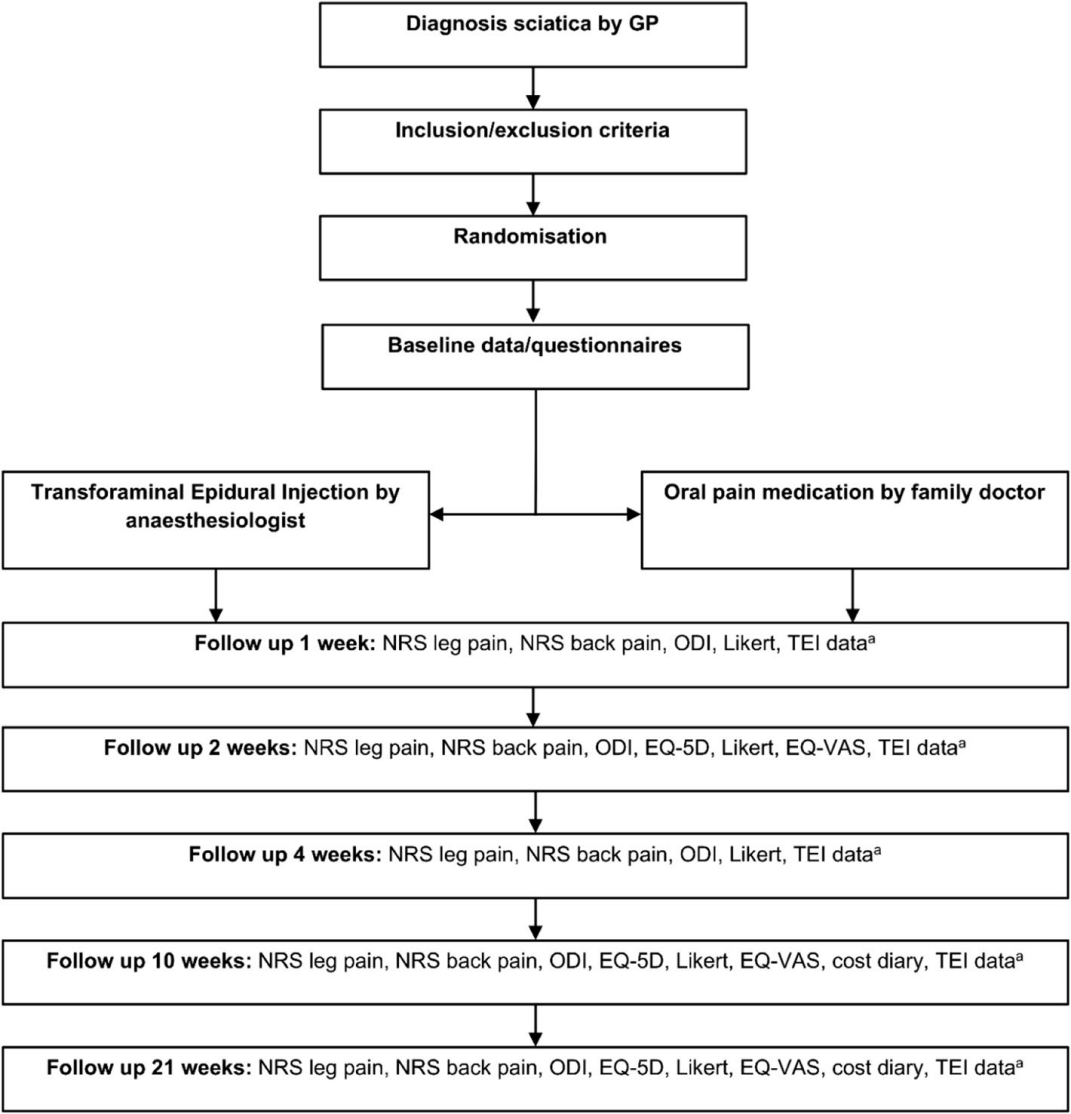
Flow diagram of TEIAS study procedures

If the patient is eligible and decides to participate, block randomization in a 1:1 allocation ratio will determine which treatment the patient will follow. Randomization is done by using web-based randomization algorithm software (Castor EDC, Ciwit B.V., Amsterdam, The Netherlands). Details on blocking of randomization are unavailable to the researcher that will perform allocation. Consequently, baseline data will be collected, questionnaires will be filled in and the patient will receive instructions for follow-up questionnaires to be filled in at home. If the patient is randomized to the intervention group, the injection will be received within 4 working days after randomization. Level of injection will be determined by the anaesthesiologist although the neurosurgeon can advise. Patients from the control group will receive oral pain medication according to the care of their GP. Patients will be evaluated at baseline and after 1, 2, 4, 10 and 21 weeks. A schematic overview of the trial is shown in Fig. 1.

Patients that are randomized to the TEI group can receive additional injections if deemed profitable by the anaesthesiologist but an injection is only repeated after a certain time interval according to anaesthesiology guidelines for usual care. For all injections, details on timing, frequency and prevailing complications will be gathered.

### Description of interventions

#### Transforaminal epidural injection (intervention group)

Patients allocated to the intervention group will receive TEI within 4 working days after randomization. Before this procedure, the patient will undergo an X-ray of the lumbar spine in order to identify any spinal abnormalities that may complicate the treatment. Administration of TEI will be performed by an experienced anaesthesiologist. The patient will lie in a prone position on the table. The skin is sterilized with chlorhexidine. Under X-ray guidance using the “tunnel vision” technique the needle is placed via a transforaminal approach in close proximity of the nerve root and contrast agent is injected to confirm correct positioning of the needle. In accordance with current Dutch anaesthesiologic guidelines, injections at L3 and below will contain 1,5 ml lidocaine 2% and 40 mg methylprednisolone acetate whereas injections above L3 will contain 1,5 ml lidocaine 1% and 10 mg dexamethasone. This is due to the possibility that particulate steroids like methylprednisolone acetate can occlude arterial blood vessels and result in an infarction of the spinal cord, the brain stem, cerebrum or cerebellum and therefore injections above L3 contain a non-particulate corticosteroid [15]. After the procedure, the patient will stay for 30 min at the recovery room for monitoring. If during the course of the study patients request additional injections, the complete procedure will be repeated for every injection.

#### Continued conservative care (control group)

The current standard for conservative care in patients with sciatica during the first 14 to 16 weeks is oral pain medication. Patients randomized to the control group can use over the counter medication or pain medication prescribed by the GP according to national guidelines. Use of pain medication will be recorded through the web-based questionnaires. If deemed necessary, the GP can additionally prescribe physiotherapy.

### Referral to neurologist or neurosurgeon

If leg pain remains severe at 2 weeks after randomization, the patient is allowed to deviate from the study protocol. The GP can refer the patient to the neurologist and the patient can be offered surgery if applicable. An NRS for leg pain of 4 or higher makes the patient eligible for surgery if the patient requests surgery. Patients are still encouraged to postpone surgery till 14–16 weeks after onset of pain. This decision is made using Shared Decision Making and it is possible that the patient is operated before this time point. However, patients that are randomized cannot receive surgery within the first two weeks, unless cauda equine syndrome or progressive paresis is reported. It is expected that during these two weeks the transforaminal epidural injection will have its effect.

### Adjuvant care

Intake of oral pain medication is allowed in addition to assigned treatment. Since it is a randomized controlled trial, it is reasonable to expect that participants in both treatment arms will demonstrate comparable variation in oral pain medication.

### Description of study parameters

Study parameters are assessed at baseline by a researcher and consequently by patients through online questionnaires at home. Data on the administration of TEI is collected by the anaesthesiologist. A summary of all study parameters and correlating follow-up moments can be found in Table 2.

**Table 2:** Data collection schedule

	Baseline	Weeks of follow-up				
		1	2	4	10	21
Demographic data	✓					
NRS leg pain	✓	✓	✓	✓	✓	✓
NRS back pain	✓	✓	✓	✓	✓	✓
ODI	✓	✓	✓	✓	✓	✓
EQ-5D	✓		✓		✓	✓
Likert score		✓	✓	✓	✓	✓
EQ-VAS	✓		✓		✓	✓
Cost diary					✓	✓
TEI data^a^		✓	✓	✓	✓	✓

^a^Only collected for subjects that received TEI

#### Primary study parameter

- The primary study parameter, NRS leg pain, will be measured at all follow-up visits: at baseline and at 1, 2, 4, 10 and 21 weeks after randomization. NRS leg pain will be scored on a 0 to 10-point scale with increments of 1 point and patients are not entitled to see pain scores from previous follow-up moments. Each patient will score the NRS leg pain based on the mean leg pain over the past week.

#### Secondary study parameters

In order to assess functionality, perceived recovery, perceived general well-being and cost-effectiveness secondary study parameters will be measured.

- NRS back pain will be scored at all follow-up visits: at baseline and at 1, 2, 4, 10 and 21 weeks after randomization. NRS back pain will be measured on a 0 to 10-point scale with increments of 1 point and patients are not entitled to see pain scores from previous follow-up moments. Each patient will score the NRS back pain based on the mean back pain over the past week;
- The Oswestry Disability Index (ODI) will be measured to assess the functionality of the patient directed at walking and daily activities. The ODI will be scored on a 0 to 50-point scale at all follow-up visits: at baseline and at 1, 2, 4, 10 and 21 weeks after randomization. Patients are not entitled to see ODI scores from previous follow-up moments;
- Perceived recovery will be measured using the Likert scale. This is a 7-point scoring scale that ranges from ‘completely recovered’ to ‘worse than ever’. The Likert score will be determined at baseline and at 1, 2, 4, 10 and 21 weeks follow-up;
- As an indicator for the patients’ perspective on his or her health state, EuroQoL Visual Analogue Scale (EQ-VAS) will be determined using a 0 (as bad as death) to 100-point scale (perfect health) with increments of 1 point. This will be measured at baseline and at 2, 10 and 21 weeks follow-up;
- Data on timing of the injection (days after randomization), frequency of the injection, NRS leg pain after injection, prevailing complications and certainty of the anaesthesiologist that the patient is suffering from sciatica and that the injection has been administered at the correct lumbar level will be gathered during the total follow-up period of 21 weeks;
- For the economic evaluation (CEA), the five-level EuroQoL (EQ-5D) will be used. This questionnaire measures five aspects: mobility, self-care, daily activities, pain/discomfort, and anxiety/depression. The EQ-5D scores will be assessed at baseline and at 2, 10 and 21 weeks follow-up. In addition, cost diaries will be filled out by the patient at 10 and 21 weeks after randomization. Health care utilization including physiotherapy, visits to GP and specialists, nursing care and medication, patients costs, and absenteeism will be evaluated;

### Data collection and protection

Data will be gathered using Castor EDC, a web-based secured data-capture platform (Ciwit B.V., Amsterdam, The Netherlands). This digital system will give each patient an untraceable ID. Only the main investigator, an independent monitor from the LUMC and the Inspection Healthcare and Youth (IGJ) will have access to the key that links these IDs to the patient’s personal details. All data will be safeguarded and stored for 15 years after end of study. Patients will fill in digital questionnaires for all follow-up moments at home using the link they received in an e-mail. When the questionnaire is completed, it will automatically be processed in Castor EDC. Only members of the research team, an independent monitor from the LUMC and the Inspection Healthcare and Youth (IGJ) will have access to the final dataset.

### Sample size

It is hypothesized that patients in the intervention group will have a mean NRS for leg pain of 4.0 on a 11-point scale at two weeks after treatment with TEI and patients in the control group will have a mean NRS for leg pain of 5.5 two weeks after randomization. Based on literature review, a difference of 1.5 points on the NRS scale will be considered clinically relevant and a standard deviation of 2.6 is considered [12, 16]. Together with a power of 90% (β = 0.10) and a level of significance of 5% (α = 0.05), 64 patients per group are needed. With two study groups and an estimated loss to follow-up of 10% a total of 142 patients needs to be recruited.

### Statistical analysis

All study parameters will be analysed according to the intention-to-treat (ITT) principle.

### Demographic statistics

Demographic data on age, gender, length, weight, tobacco exposure, alcohol abuse, history of previous radicular symptoms in the legs and usage of pain medication will be reported using mean and standard deviation or median and ranges if distribution is skewed. Presuming distribution is normal age, length and weight will be compared between groups using t-tests. Gender, tobacco exposure, alcohol abuse, history of previous radicular symptoms in the legs and usage of pain medication will be assessed using the Chi-square test.

#### Primary analysis

- The primary study outcome, the average score on the NRS leg pain scale at two weeks, will be compared between groups utilizing the unpaired t-tests.

#### Secondary analysis

- The average score on the NRS leg pain scale at 4 weeks will be compared between groups utilizing the unpaired t-tests.
- The absolute decrease of NRS leg pain, NRS back pain, ODI and increase of EQ-5D will be analysed using t-tests for the period from baseline to 2 weeks follow-up and from baseline to 4 weeks follow-up;
- Perceived recovery Likert scores will be dichotomized: 1–2 will be considered a success and 3–7 will be considered no success. Consequently, data will be compared using the Chi-square test at 2 and 4 weeks after randomization or injection to 10 and 21 weeks after randomization;
- The success data from the 2-week follow-up will be correlated to the success data at 14 weeks and 26 weeks using Chi-square tests to make predictions. In order to correlate 2-week and 4-week success data to the absolute data for NRS leg pain, NRS back pain, ODI and EQ-5D at 10 and 21 weeks, logistic regression analysis will be used;
- Baseline data will be correlated to the 2-week success data after treatment with TEI using t-tests for numerical variables and Chi-square test for categorical variables;
- The cost-effectiveness analysis will be evaluated from a healthcare perspective (the costs per extra patient with symptom relief) and a cost-utility analysis from a societal perspective (costs per Quality Adjusted Life Year (QALY)). Both analyses will be trial-based, with a time horizon of 21 weeks. Use of health care and productivity will be valued according to the Dutch guidelines. QALYs will be assessed as the area under the utility curve based on the Dutch tariff for the EQ-5D. For sensitivity analysis, QALYs will be determined using the VAS for quality of life with power transformation. The average costs and patient outcome will be compared according to intention-to-treat, using net-benefit analysis, and using multiple imputation to account for missing data;

### Withdrawal of subjects

Subjects can withdraw from the study at any time for any reason without consequences and will not be replaced. Subjects that refuse TEI after randomization to the TEI group, will be considered as cross-over subjects. Patients that withdraw during the study will be considered lost-to-follow-up patients. The study investigator can decide to withdraw a subject for urgent medical reasons.

### Study monitoring

#### Data monitoring

Data monitoring will be performed by an independent monitor from the LUMC twice per year and will oversee the handling, safeguard and storage of data in accordance with the LUMC protocol and Good Research Practice (GRP) guidelines. No data monitoring committee is involved since TEI is considered usual care and risks are considered to be moderate.

#### Adverse event management

Adverse events (AEs) are defined as any undesirable experience occurring to a subject during the study, whether or not considered related to TEI. All adverse events reported spontaneously by the subject or observed by the investigator or his staff will be recorded. Adverse events from treatment with TEI are rare. The most common AEs reported in literature are transient exacerbation of pain (2.4%), pain at site of injection (1.1%) and accidental dural puncture (2.3%) [17].

Serious adverse events (SAEs) are defined as any untoward medical occurrence or effect that result in the subject’s death or is life threatening, require hospitalisation or prolongation of existing inpatients’ hospitalisation, result in persistent or significant disability or incapacity, is a congenital anomaly or birth defect or any other important medical event that did not result in any of the outcomes listed above caused by medical or surgical therapy, but could have been based upon appropriate judgement by the investigator. Elective hospitalization will not be considered as a serious adverse event. SAEs are reported by the investigator through the online web portal *ToetsingOnline* to the accredited Medical Ethics Committee (MEC) that approved the protocol within one week of first knowledge of SAEs that result in death or are life threatening followed by a period of maximum of 8 days to complete the initial preliminary report. All other SAEs will be reported within a period of maximum 15 days after first knowledge of the events.

All AEs will be followed until they have subsided or until a stable situation has been reached. Dependent of the kind of event, additional tests or medical procedures may be required for follow-up, and the subject may be referred to the general practitioner or a medical specialist.

In literature arachnoiditis and ‘nervous system disorder’ have been described as major complications. Despite the lack of exact data, these complications are considered to be rare [17].

#### Interim analysis

The investigator will annually submit a summary of the progress of the trial to the accredited MEC. Information will be provided on the date of inclusion of first subject, total number of subjects included to that point, total number of subjects that have completed the trial, serious adverse events, other problems and amendments. Additionally, the independent data monitor and the Inspection Healthcare and Youth (IGJ) will have access to interim analyses.

#### Premature termination of study

In case the study is ended prematurely, the sponsor will notify the accredited MEC within 15 days, including the reasons for the premature termination.

#### Dissemination of results

The results from the TEIAS Trial will be published in international peer reviewed neurological and neurosurgical related journals. Results will be presented at national and international spine and pain congresses. Furthermore, the results can influence the usual care in the Netherlands and will subsequently be offered to be considered by (inter) national guideline-authors for treatment of sciatica.

## Discussion

The current guidelines on treatment of sciatica state that a period of conservative therapy up to 14–16 weeks is preferred and these guidelines are based on the results from the Sciatica trial by Peul et al. During this trial, 40% from the group that received continued conservative care crossed-over to the surgical group with a mean delay of 15 weeks [8]. The TEIAS-trial is focused on finding an appropriate type of conservative treatment to bridge the time during this period of conservative care in the acute phase of sciatica.

For the TEIAS study it is presumed that eligible patients will have an average duration of symptoms of 5 weeks when randomized to either the treatment arm or control arm. Therefore, follow-up at 10 weeks will correspond with the 14–16 week time point from the Sciatica trial. In the same manner, the 21 week follow-up moment will be similar to the 26 week time point in the Sciatica trial at which surgical intervention did not yield a superior patient outcome compared to conservative therapy. Therefore, the short-term effect of TEI will be evaluated at 1, 2 and 4 weeks and long-term evaluation will be performed at 10 and 21 weeks.

The burden from the pain and resulting immobility during the acute phase of sciatica have been a challenge and an adequate routine treatment strategy to alleviate symptoms during this period of ‘wait-and-see’ is lacking. TEI has often been proposed as an option to reduce pain and inflammation caused by mechanical compression of the nerve root and literature has demonstrated this treatment to be efficacious in part of the sciatica patients [18–21]. However, only few studies have investigated the use of TEI specifically in patients with acute sciatica (< 8 weeks). One study randomized 63 patients with a duration of symptoms between 2 and 4 weeks to either TEI or usual conservative care [11]. The TEI group experienced a greater improvement in functionality and back pain, but differences between groups were small. However, the effect of the intervention group was averaged over both responders and non-responders. Therefore, it is plausible that if the effect had been averaged only over the responders a clear effect could have been found for these patients. Moreover, the epidural injection lacked an analgesic medicament, baseline pain scores for back and leg as well as Roland Disability score were lower in the control group, and size of patient groups were small. Another study that studied TEI in the acute phase of sciatica found that 66% of patients were considered responders within hours after an injection. The VAS score for leg pain lied around 30 mm on a 100 mm scale during the first 14 days for this group. For the non-responders the VAS leg pain score remained around 55 mm on average during these two weeks [12].

A Dutch study by Spijker-Huiges et al. investigated the effect of epidural steroid injections containing 80 mg of triamcinolone in normal saline in acute sciatica patients (2–4 weeks of symptoms) [22]. They compared this intervention in addition to usual care to usual care alone similarly to our study protocol. Results showed a small but clinically irrelevant difference between groups, but cost-utility analysis demonstrated that adding epidural steroid injections decreased total health care costs for these patients. However, despite randomization of patients baseline characteristics differed statistically significantly between groups and current anaesthesiologic guidelines advise injections with dexamethasone or Depo-Medrol instead of triamcinolone which was administered in the aforementioned study. Additionally, steroids were administered through a translaminar approach in contrast to the currently preferred transforaminal technique which will be used in our study. Furthermore, patients allocated to the treatment group received only one epidural injection. Part of these patients had a positive response to the injection but the effect deteriorated after several weeks. Therefore, additional injections might be suitable for this group of patients and in our study patients can opt for further treatment with TEI if the anaesthesiologist considers this step to be advantageous.

Due to the pragmatic nature of this study patients suffering from sciatica are directly referred by the GP to the anaesthesiologist without interference of any type of imaging. With the current organization of usual care it takes several weeks before the patient has visited the neurologist, undergone MRI examination and received TEI. In order to minimize this waiting time only a neurosurgeon will shortly examine the patient before inclusion to confirm that the patient is suffering from sciatica. This means that determination of the herniated disc level is based on medical history taking and physical examination. The neurosurgeon will advise the anaesthesiologist on the affected nerve root level, but if a disagreement exists the anaesthesiologist has the final decision on the level to be treated. In order to determine whether uncertainty with regard to the level of injection may play a role in the effectiveness of TEI the anaesthesiologist is asked to express his confidence in the correctness of the diagnosis and of the treated disc level.

An interesting feature of TEI that has only been studied before to a limited extent is its predictive capability on long-term patient outcome. In theory, if such predictive power of TEI exists, patients that will not do well on the long term based on the short-term effect of TEI can be offered surgery at an earlier time point. For instance, a patient could become eligible for surgery after two weeks based on the two-week TEI outcome and would not have to wait until the 14–16 week time point. Joswig et al. addressed this predictive feature in a prospective study [12]. The results demonstrated that a decrease of less than 50% in leg pain within one week indicated an unfavourable outcome at one month and therefore other treatment options should be considered. Furthermore, data suggested that prediction of the 3-month outcome might be possible which makes this a promising concept [13]. A study by El-Yahchouchi et al. investigated the predictive power of TEI for long-term outcome in patients with radicular pain, regardless of the presence of radiculopathy [14]. They assessed the effect of the epidural injection immediately after administration, at 2 weeks and at 2 months. Results showed that patient outcome at two months, the long-term effect, was not associated with immediate pain relief. However, the long-term effect was strongly related to pain relief at 2 weeks. They argued that the delayed effect from the anti-inflammatory steroid agent was not observable directly post-injection, but only established thereafter and thus was observable at the two-week follow-up moment. This is an indication that a predictive value might exist.

### Trial status

The trial is conducted according to the protocol, version 4.0 (14 February 2019). Recruitment of participants is intended to start June 2019 and is expected to be completed no later than July 1, 2020.

## Data Availability

Data will be available upon request. Requests can be made with the corresponding author.

## Abbreviations

AE: Adverse event
CEA: Cost-effectiveness analysis
EQ-5D: EuroQoL-5 Dimension
EQ-VAS: EuroQol Visual Analogue Scale
GP: General practitioner
GRP: Good research practice
IGJ: Inspection for Healthcare and Youth
ITT: Intention-to-treat
LUMC: Leiden University Medical Center
MEC: Medical Ethical Committee
MRI: Magnetic resonance imaging
NRS: Numerical rating scale
ODI: Oswestry disability index
QALY: Quality adjusted life year
RCT: Randomized controlled trial
SAE: Serious adverse event
SG: Spaarne Gasthuis
TEI: Transforaminal epidural injection
TEIAS: Transforaminal epidural injection in acute sciatica
VAS: Visual analogue scale
